# Carry-Over Factor of Zearalenone in the Roof of the Third Ventricle of the Brain and Selected Skeletal Muscles During Low-Dose Zearalenone Mycotoxicosis in Prepubertal Gilts

**DOI:** 10.3390/toxins18050224

**Published:** 2026-05-08

**Authors:** Magdalena Gajęcka, Łukasz Zielonka, Maciej T. Gajęcki

**Affiliations:** Department of Veterinary Prevention and Feed Hygiene, Faculty of Veterinary Medicine, University of Warmia and Mazury in Olsztyn, Oczapowskiego 13, 10-718 Olsztyn, Poland; lukasz.zielonka@uwm.edu.pl (Ł.Z.); mgajecki48@gmail.com (M.T.G.)

**Keywords:** zearalenone mycotoxicosis, roof of the third cerebral ventricle, skeletal muscle, prepubertal gilts

## Abstract

The aim of this study was to determine whether exposure to low doses of zearalenone (ZEN) over a period of six weeks affects the values of the carry-over factor (COF) of ZEN in the roof of the third cerebral ventricle (RTCV) and in selected skeletal muscles (*longissimus* and *quadriceps*) in prepubertal gilts. The study was conducted on 60 clinically healthy prepubertal gilts with an initial body weight (BW) of 14.5 ± 2 kg. Gilts were randomly assigned to a control group (group C; *n* = 15) and three experimental groups (ZEN5, ZEN10, and ZEN15; *n* = 15 each). Groups ZEN5, ZEN10, and ZEN15 were administered ZEN *per os* at doses of 5 µg/kg BW, 10 µg/kg BW, and 15 µg/kg BW, respectively. Group C animals were orally administered a placebo. Tissue samples (brain and skeletal muscles) were collected post-mortem for toxicological analyses on exposure days 7 (D1), 21 (D2), and 42 (D3). The concentrations of ZEN and its metabolites, α-zearalenol (α-ZEL) and β-zearalenol (β-ZEL), were determined in the collected samples. All examined tissues contained the parent compound, but ZEN metabolites were not detected in any of the samples. The absence of ZEN metabolites may have resulted from a physiological deficit of estradiol (E_2_) and, consequently, testosterone (T) and progesterone (P_4_) in prepubertal gilts. Low-dose ZEN mycotoxicosis led to a persistent presence of ZEN in the RTCV (COF from 1 × 10^−6^ on D1 to 7 × 10^−7^ on D3) and somewhat lower ZEN levels in skeletal muscles (COF from 8 × 10^−6^ on D1 to 6 × 10^−7^ on D3). The presence of ZEN in the RTCV confirms that it crosses the blood–brain barrier and may therefore participate in the hormonal homeostasis of the brain.

## 1. Introduction

In toxicology, the carry-over factor (COF) refers to residues from previous exposure, treatment, or dosing that persist and impact subsequent responses, often acting as a confounding factor in research or potentially contributing to cumulative toxicity. In biological sciences, the COF occurs when previous exposure to a given substance affects an organism’s present condition or traits. A correct interpretation of the COF rests on the premise that a high COF implies a strong relationship between earlier and subsequent stages that substantially shapes current outcomes, whereas a low COF suggests a limited influence of past events on the present. For example, feed availability in early life may act as a COF, whereas suboptimal conditions can delay growth and potentially affect the physiological and somatic development of young organisms [1].

Zearalenone (ZEN) mycotoxicosis is a form of poisoning that results from ingesting feed or food contaminated with ZEN, an estrogen-like mycotoxin produced by *Fusarium* mold fungi that contaminate crops such as maize, wheat, barley, oats, and rice. Owing to its structural similarity to estrogens, ZEN can bind to estrogen receptors (ERs) in various organs [2,3,4], including the liver and the gastrointestinal tract [5,6] as well as in muscle tissue [7,8]. Zearalenone remains highly stable in stored foods and is resistant to processing, which increases the risk of exposure through the diet [9]. The consumption of ZEN-contaminated food can weaken the immune system [10], modulate inflammatory responses, and disrupt DNA integrity [11]. The International Agency for Research on Cancer has classified ZEN as a group 3 carcinogen due to limited evidence in animals and insufficient evidence in humans [12,13,14]. Zearalenone and its metabolites, particularly α-zearalenol (α-ZEL) and β-zearalenol (β-ZEL), may accumulate in muscle tissue after consumption [6,7]. The presence and levels of these compounds in muscles should be monitored to ensure food safety because they are regarded as substances that disrupt hormonal system function and pose a potential threat to human and livestock health [3,15,16,17].

The roof of the third cerebral ventricle (RTCV) is composed primarily of the fornix and the *velum interpositum*, and it anteriorly connects to the lateral ventricles through the foramina of Monro. All of these structures are critical because they play important roles in the central nervous system [18]. The choroid plexus (ChP) in the RTCV is a key component of the blood-cerebrospinal fluid barrier and an important immunological control interface of the brain. Recent studies have suggested that changes in ChP volume are associated with pathological processes in the brain [19]. Local inflammatory cell infiltration of varying etiology contributes to ChP enlargement. These conditions lead to functional changes in the ChP, resulting from tissue damage, blood–cerebrospinal fluid barrier dysfunction, and impaired fluid transport [20]. As a critical modulator of the central nervous system, the ChP should be regarded as one of the circumventricular organs due to its location at the brain periphery near the ventricles, high vascularization, a functional role in endocrine signaling, production of proteins secreted into the cerebrospinal fluid, and presence of fenestrated vessels [18,21].

Skeletal muscles constitute the largest component of the locomotor system. They serve as a major protein reservoir, act as the primary site of glucose metabolism, and account for approximately 40% of total body mass. Skeletal muscles are voluntarily controlled and are responsible for body motion, maintenance of posture, and heat generation through contraction and relaxation [22]. Genetic research facilitates the elucidation of regulatory mechanisms that govern the growth and development of porcine skeletal muscles, thereby contributing to the accelerated improvement of performance traits in pork production. Muscle development proceeds in two consecutive stages: formation of muscle fibers before birth and hypertrophy of muscle fibers after birth. The adult stage, which is part of the second phase, coincides with the period of muscle growth and development [23], corresponding to somatic development. Moreover, skeletal muscles have also been identified as active secretory organs. Muscle fibers synthesize and secrete various bioactive cytokines and humoral factors (myokines), which are involved in numerous intercellular signaling processes via endocrine, paracrine, and autocrine pathways. These properties play a pivotal role in regulating physiological and pathological processes [22]. Specific damage to skeletal muscles and changes in the composition of muscle fibers resulting from ZEN exposure remain insufficiently investigated [16]. Damiano et al. [24] found that natural exposure to ZEN induced slight changes in the morphology and diameter of muscle fibers in boars from southern Italy. The cited study suggests that animals exposed to ZEN may exhibit skeletal muscle damage. According to the above authors, direct ZEN-mediated pathological changes in skeletal muscles are difficult to identify due to the complex interplay between multiple mycotoxins in mold-contaminated cereal crops and derived products.

It was hypothesized that ZEN, a compound with estrogen-like and pleiotropic activity [15], may influence the muscle–brain axis [8]. This interaction is mediated by ERs, which are present in both the brain and skeletal muscles [2,3,14]. In addition, ZEN crosses the blood–brain barrier [4,8] and accumulates in skeletal muscles [16,25,26]. In the brain, particularly within the third ventricle, ZEN participates in neuroendocrine–immune regulation [19,21]. Furthermore, this mycotoxin induces quantitative changes in the production of gonadotropic [1] and steroid hormones [4], and is therefore considered an epigenetic factor in maturation [22,27]. In turn, skeletal muscles play a key role in whole-body energy metabolism [16,28]. In mammals, energy homeostasis [29] is regulated by the central nervous system through the detection of circulating factors such as myokines [8,22], which encode physiological states and elicit responses via efferent signaling mediated by endocrine or neuronal cells [30]. After crossing the blood–brain barrier, myokines may also act directly on brain tissue [31,32]. Physical activity is a key physiological stimulus [33], and prepubertal gilts are young and highly active animals [22]. Tanycytes, specialized cells lining the third ventricle, also play an important role in the regulation of feeding behavior, glucose homeostasis, and energy expenditure. These cells are strategically located at the interface between the brain and peripheral organs, facilitating bidirectional communication.

This likely bidirectional interaction between skeletal muscles and the endocrine system contributes to a better understanding of both clinical conditions and muscle function [8], lending support to the concept of the muscle–brain axis [8]. These considerations provided the rationale for the present study.

The aim of this study was to determine whether exposure to low doses of ZEN administered orally to prepubertal gilts for six weeks (Minimal Anticipated Biological Effect Level, MABEL [5 μg ZEN/kg BW—group ZEN5]; highest No Observed Adverse Effect Level, NOAEL [10 μg ZEN/kg BW—group ZEN10], and Lowest Observed Adverse Effect Level, LOAEL [15 μg ZEN/kg BW—group ZEN15]) affects COF values and the concentrations of ZEN, α-ZEL, and β-ZEL in the RTCV and selected skeletal muscles.

## 2. Results

Clinical signs of zearalenone mycotoxicosis were not observed during the experiment. However, changes in specific tissues or cells were frequently observed in endocrine values and metabolic profile analyses of samples collected from the same animals. The results of these analyses were published in different papers [3,4,5,6,7,15,17,29,34,35].

### 2.1. Experimental Feed

The analyzed feed did not contain mycotoxins, or its mycotoxin content was below the limit of detection (LOD). The concentrations of modified and masked mycotoxins were not analyzed.

### 2.2. Concentrations of ZEN and Its Metabolites

The concentrations of both ZEN metabolites were below the LOD in all analyzed tissues (Table 1).

An analysis of Table 1 data indicates that ZEN concentrations in the examined tissues varied substantially across groups and tissues as well as on different exposure dates. Significant differences at *p* ≤ 0.05 (*) were observed only in the *longissimus* muscle between group ZEN5 and group ZEN15 on D1 and D2 (difference of 4.45 and 3.89 ng/g, respectively).

In the RTCV and the *quadriceps* muscle, no significant differences in ZEN concentration were observed between groups or sampling dates (Table 1). The examined parameter was highest in the *quadriceps* muscle in group ZEN5 on D1 and D3. In groups ZEN10 and ZEN15, ZEN concentrations were highest in the *longissimus* muscle on all sampling dates.

An analysis of mean ZEN levels in the studied tissues on different exposure dates indicates that ZEN concentration remained consistently lowest in the RTCV throughout the entire exposure period. In turn, ZEN concentration was significantly highest in the *longissimus* muscle on all dates, with a minor decreasing trend observed over time.

### 2.3. Carry-Over Factor

An analysis of COF values (Table 2) indicates that RTCV samples consistently exhibited the lowest COF values (for negative values, a lower magnitude reflects greater accumulation of ZEN in the analyzed tissues) across all experimental groups and exposure dates. A slight decrease in ZEN transfer efficiency was observed only on exposure date D3 (groups ZEN10 and ZEN15).

The highest ZEN COF values were observed in the *longissimus* muscle compared with the remaining analyzed tissues (RTCV and *quadriceps*) (Table 2). The examined parameter was highest on exposure date D1, particularly in group ZEN15 (5 × 10^−6^), and it decreased on subsequent exposure days (by 50% on D2 and by 75% on D3). An analysis of COF values across exposure dates further indicated the lowest values in group ZEN5 and markedly higher values in groups ZEN10 and ZEN15.

In the *quadriceps* muscle, ZEN COF values differed across experimental groups (Table 2). In general, COF values decreased over time during continued exposure, excluding in group ZEN5. In this group, COF values on exposure dates D1 and D3 were nearly identical and markedly higher than those observed on D2.

### 2.4. Null Hypothesis Significance Testing

The results are tested against zero to assess whether they are significantly different from the assumption that there is no effect, relationship, or difference (the null hypothesis). The interpretation depends on the *p*-value: if *p* is lower than the predetermined significance level (usually 0.05), the null hypothesis is rejected, indicating that the observed results are unlikely to have occurred by chance. A more stringent significance level (such as 0.01) is set to increase the statistical rigor of the analysis. In this case, the null hypothesis is rejected with greater caution, but the likelihood of obtaining significant results is also reduced.

**Figure 1:**
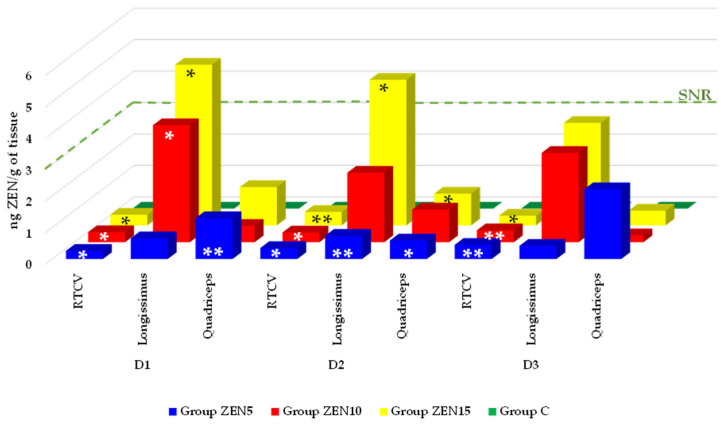
Comparison of the $\overset{-}{x}$ of ZEN concentrations in each experimental group relative to zero (group C) on all exposure dates. Key: RTCV—roof of the third cerebral ventricle; D1—exposure day 7; D2—exposure day 21; D3—exposure day 42; Experimental groups: Group ZEN5—5 µg ZEN/kg BW; Group ZEN10—10 µg ZEN/kg BW; Group ZEN15—15 µg ZEN/kg BW; Group C (control)—0 µg ZEN/kg BW, with no ZEN exposure; n = 5 in each group and on each date; SNR—signal-to-noise; ratio = 3. Cut-off points were established at the significance levels of * *p* ≤ 0.05 and ** *p* ≤ 0.01.

When ZEN concentrations in the RTCV were tested against zero (i.e., group C), the results were statistically significant (Figure 1). On exposure date D1, significant differences at *p* ≤ 0.05 were observed in all experimental groups (0.26, 0.32, and 0.34 ng ZEN/g in ZEN5, ZEN10, and ZEN15, respectively). On D2, significant differences at *p* ≤ 0.05 were found in groups ZEN5 and ZEN10 (0.35 and 0.31 ng ZEN/g, respectively), and a significant difference at *p* ≤ 0.01 was noted in group ZEN15 (0.43 ng ZEN/g). On D3, a significant difference at *p* ≤ 0.05 was observed in group ZEN 15 (0.31 ng ZEN/g), and significant differences at *p* ≤ 0.01 were found in groups ZEN5 and ZEN10 (0.45 and 0.38 ng ZEN/g, respectively).

In the *longissimus* muscle (Figure 1), on exposure date D1, significant differences at *p* ≤ 0.05 were noted only in groups ZEN10 and ZEN15 (3.72 and 5.11 ng ZEN/g, respectively). On D2, a significant difference at *p* ≤ 0.05 was determined in group ZEN15 (4.63 ng ZEN/g), and a significant difference at *p* ≤ 0.01 was found in group ZEN5 (0.74 ng ZEN/g). No significant differences were observed on D3.

In the *quadriceps* muscle, a significant difference at *p* ≤ 0.01 was noted on exposure date D1 only in group ZEN5 (1.30 ng ZEN/g) (Figure 1). On D2, significant differences at *p* ≤ 0.01 were observed in groups ZEN5 and ZEN 15 (0.59 and 1.01 ng ZEN/g, respectively). No significant differences were found on D3.

### 2.5. Summary of Results

The present findings provide a basis for subsequent research into the effects of very low ZEN doses on the macroorganism. In prepubertal gilts, a specific relationship may exist between mycotoxin levels in the feed and the microenvironment of the RTCV and the examined skeletal muscles (*longissimus* and *quadriceps*). Preliminary analysis indicates marked inter-individual variability in ZEN concentrations as well as the absence of major ZEN metabolites in the studied tissues. This experiment confirmed that the applied chromatographic method is suitable for precise quantitative analysis, enabling assessment at both individual and population levels.

## 3. Discussion

The results of quantitative analyses examining the levels and carry-over factor of ZEN, α-ZEL, and β-ZEL in selected skeletal muscles (*longissimus* and *quadriceps*) and in the RTCV pose interpretational challenges. Due to the lack of similar studies in the literature, these findings should be extrapolated with caution.

Estrogen receptors [2,3,5,14] constitute the primary link underlying the effects of exposure to mycoestrogens such as ZEN. Already at the turn of the 20th and 21st centuries, ERs were suggested to play a role in the physiology of skeletal muscles [20,22,36] and the brain [4,37,38]. The authors’ previous studies suggest that some mycoestrogens, including ZEN and deoxynivalenol (DON), bind to ERs with a preference for ERα [3,5,38]. These studies reported on ER expression in the smooth muscles of the reproductive tract or the intestines, and in the nervous system of prepubertal gilts [4]. In contrast, knowledge regarding ER expression in skeletal muscles remains limited. The available data suggest a balance between ERα and ERβ in this tissue [26].

### 3.1. Zearalenone

Until recently, there was no evidence that ZEN and its metabolites accumulate in skeletal muscles or that skeletal muscles could be a target tissue for ZEN [16]. The present study provides evidence that such accumulation is possible.

#### 3.1.1. Pleiotropic Effects of ZEN

The administered doses were very low, not only in group ZEN5 (MABEL) but also in groups ZEN10 (NOAEL) and ZEN15 (LOAEL). This observation poses a challenge, as a deficiency of endogenous steroids is a normal physiological state in prepubertal gilts, whereas exposure to ZEN disrupts hormonal homeostasis [4,17].

Zearalenone concentrations in the analyzed tissues as well as the absence of ZEN metabolites on all exposure dates (Table 1) elucidate ZEN distribution and biotransformation processes in prepubertal gilts. It follows that the orally administered ZEN doses were insufficient to induce hyperestrogenism but supplemented physiological deficits in endogenous steroid hormones [39].

The observed toxicokinetic profile of ZEN (absorption, distribution, metabolism and excretion) should be interpreted differently. In prepubertal gilts, ZEN biotransformation (functionalization and conjugation) occurs much more slowly than ZEN binding to ERs [40]. This suggests that the administered doses of ZEN supplemented physiological deficits in endogenous steroid hormones (Table 1) in these animals [41] and raises the question of whether ZEN-mediated interference with physiological endocrine pathways in prepubertal gilts is a neutral process. This is not obvious—firstly, by binding to ERs, ZEN modifies endocrine homeostasis and leads to dysfunctions in steroidogenesis processes, including an increase in E_2_ levels that decreases P_4_ and T concentrations [4]. Secondly, the resulting hormonal changes inhibit the somatic development of reproductive tract tissues [17,29].

Several hypotheses may be proposed to explain the absence of ZEN metabolites. First, this finding may reflect a physiological adaptation in prepubertal females that is potentially related to low levels of endogenous steroid hormones [17]. Under such conditions, ZEN metabolites may partially compensate for a deficiency of endogenous steroid hormones that are required for normal physiological function [2,4]. According to the cited authors, the biotransformation of ZEN in the intestines and bloodstream may proceed more slowly than ZEN binding to steroid receptors. Second, ZEN is metabolized mainly in the liver, and hepatoxicity is one of the most common adverse effects [42,43]. Moreover, Sayed et al. [44] and Balló et al. [45] demonstrated that parental exposure to ZEN may induce integrated liver pathophysiology in F1 offspring, leading to metabolic dysregulation and reduced detoxification capacity toward ZEN [9]. Third, the standard deviations were widely dispersed relative to the mean concentrations and exceeded one-third of the mean values, which points to high variability in the dataset [46,47]. This variability may be associated with episodic and rapid binding of ZEN to ERs in prepubertal gilts that occurred more quickly than the biotransformation of this undesirable substance. These factors, acting individually or in combination, may have influenced the concentrations of ZEN and its metabolites in the peripheral blood of the study groups, thus reflecting this mycotoxin’s multifaceted biological activity [15].

#### 3.1.2. Neuroendocrine Activity of ZEN

Neuroendocrine activity refers to the ability of specialized cells to produce, store, and secrete hormones and biogenic amines in response to interoceptive signals, thereby regulating the overall homeostasis. Research has shown that ZEN adversely affects: (i) neuroendocrine coordination of reproductive function in prepubertal gilts [4] and (ii) the RTCV, in particular ChP, which participates in neuroendocrine-immune regulation of the brain [19,21]. This implies that ZEN may contribute to neuroinflammatory processes in the brain [20] as well as to myolysis at different stages of exposure [16].

#### 3.1.3. Adverse Effects of ZEN Exposure

The mechanisms by which estrogens affect muscle mass are also important. The molecular crosstalk involving estrogen or estrogen-like compounds such as ZEN (mycoestrogen) influences the cellular regulation of gene expression among signaling pathways, including apoptotic signaling, alterations in contractile proteins, and the maintenance of muscle satellite cells [16,48]. In addition, skeletal muscle mitochondria and stem cells specific to skeletal muscles contain ERs that respond to these compounds [49]. The current study demonstrated (Table 1) that ZEN concentrations in skeletal muscles were higher in the first three weeks of exposure (up to the experimental day 21). This means that ZEN accumulates in skeletal muscles that constitute one of the target tissues. As a result, ZEN acts as a predisposing factor for oxidative stress (unlike estrogen), leading to impaired cell viability [50,51]. This disturbance reflects stress-related activation, accompanied by the excessive production of reactive oxygen species (ROS), which disrupts antioxidant homeostasis [10], impairs cellular function, and ultimately leads to apoptosis or necrosis [52]. This suggestion is supported by the findings of Li et al. [16] who documented that ZEN induces oxidative stress, decreases the mean area and diameter of muscle fibers, and contributes to myolysis, a pathological condition involving the destruction of muscle fibers.

#### 3.1.4. Energy Homeostasis

Both estrogen and ZEN are implicated in glucose uptake. Skeletal muscles are the key sites of whole-body energy metabolism [15,28,29]. In turn, whole-body energy homeostasis requires precise coordination between dietary nutrient intake and energy expenditure. In mammals, energy homeostasis is maintained by the central nervous system, which identifies circulating factors (such as myokines [8,22]) and interoceptive signals that encode biological states and elicit the appropriate responses via endocrine or neural efferent signaling pathways [31]. Locomotion is the most important physiological stimulus [33], and prepubertal gilts are young and highly active animals [22].

### 3.2. Carry-Over Factor

Carry-over factor values refer to the transfer of undesirable substances, such as ZEN, from contaminated feed to animal tissues and by-products [53]. Zearalenone is one of the seven most widely investigated mycotoxins in studies analyzing the transfer of undesirable compounds to food [3]. It should be noted that lower values at negative exponents reflect greater saturation of the analyzed tissues with ZEN (Table 2) [54]. The pattern of ZEN accumulation differed slightly between the *quadriceps* and *longissimus* muscles (Table 2). For example, COF values for the *quadriceps* muscle were highest on exposure dates D1 and D3 (1 × 10^−5^ on both dates) in group ZEN5, whereas COF values for the *longissimus* muscles were highest on D1 and D2 (2 × 10^−5^ and 1 × 10^−5^, respectively) in group ZEN15. This implies that the highest ZEN concentrations in the studied tissues occurred on different dates and at different exposure doses [24].

#### 3.2.1. Transfer Effects

The key aspects of carry-over effects should be examined from multiple perspectives, including the integration of different life stages, environmental influences, and effects on physiological condition. Regarding the first aspect, carry-over effects act as links between different development stages in prepubertal gilts, such as prepubescence and pubescence. The present experiment was conducted on prepubertal gilts administered very low ZEN doses, such as the MABEL dose in group ZEN5. This is due to the fact that as a mycoestrogen, ZEN does not conform to the prevailing dose–response paradigm. The low-dose hypothesis, especially in relation to hormonally active compounds, challenges this concept [40] and does not provide evidence for a hormetic effect [55]. From a medical perspective, low but measurable doses of stressors or toxic substances may exert beneficial effects by stimulating repair, adaptive, and protective processes [17]. However, this relationship is highly problematic because the non-linear dose–response pattern precludes a simple monotonic extrapolation or meta-analysis of risk assessment based on laboratory data. In addition, clinical effects observed at high doses cannot be reliably extrapolated to scenarios involving chronic low-dose exposure. The above explains why ZEN exerts indirect suppressive effects on progesterone (P_4_) and testosterone (T) levels [56]. This plays an important role in autoimmune disorders [57] as well as in pork processing (as ZEN delays sexual maturation processes in gilts) [4,58].

Other aspects that contribute to the interpretation of COF and warrant further analysis are environmental conditions combined with effects on physiology. They may act as stressors that carry effects from earlier life stages into later periods, leading to adverse outcomes or increased tolerance. In prepubertal gilts, ZEN exposure can lead to reversible or irreversible steroid hormone homeostasis as well as abnormalities in the synthesis of neuronal factors and enzymes in brain neurons [59].

#### 3.2.2. Reproductive Effects

In pigs, including prepubertal gilts, the control of reproductive functions relies on intricate regulatory networks that integrate peripheral signals with internal signals and affect brain regulatory centers such as the hypothalamic–pituitary–gonadal (HPG) axis [1,60]. In turn, the impaired reception of E_2_ signaling due to the binding of ZEN and/or its metabolites to ERs suppresses the biosynthesis and release of the two gonadotropins—the luteinizing hormone (LH) and the follicle-stimulating hormone (FSH) [1,61,62]—with a concomitant reduction in LH pulse amplitude [27,63]. This observation is reflected by the COF values presented in Table 2. On all exposure dates, COF values in the RTCV were lowest in group ZEN5 and much higher in the other experimental groups (Table 2), but ZEN saturation levels were inversely proportional due to negative exponents. Lower COF values (in response to the MABEL dose) can exert a small but persistent influence on the present or future endogenous homeostasis of steroid hormones [4,56]. In contrast, in groups ZEN10 (NOAEL) and ZEN15 (LOAEL), higher COF values cause an increase in exposure levels and have a stronger impact on the current physiological state, as determined by new environmental factors.

#### 3.2.3. Potential Role of ZEN in the RTCV

In the RTCV, ZEN levels were fairly stable on exposure dates D1 and D2, regardless of the exposure dose. Zearalenone accumulation was lower only on date D3 in groups ZEN10 and ZEN15 (Table 2). In light of these findings, it can be hypothesized that ZEN, an endocrine-disrupting chemical [40], increases the expression of ERs, thus delaying the synthesis and secretion of FSH in the porcine pituitary gland [3,5,64]. Therefore, ZEN suppressed the secretion of FSH, which inhibits the production of sex hormones such as E_2_, P_4_, and T [17]. This leads to a shift in steroid metabolism towards increased conversion to E_2_. These processes are accompanied by increased feed intake and enhanced energy storage, as reported by Gajęcka et al. [4].

### 3.3. Null Hypothesis Significance Testing

Null hypothesis significance testing has received considerable criticism in recent years, but it remains the mainstay of statistical inference [65]. The results are tested against zero to determine whether they diverge significantly from the assumption that there is no effect, relationship, or difference (the null hypothesis). The significance level should be regarded as a cut-off value. The null hypothesis should be rejected if the *p*-value, defined as the probability of observing an effect that is at least as extreme as the effect observed under the null hypothesis, is less than the significance level. This suggests that there is sufficient evidence to infer the presence of an effect or association [66]. In turn, if the *p*-value is greater than the significance level, the null hypothesis cannot be rejected, indicating that there is no sufficient evidence to support a statistically significant effect. Based on these suggestions, cut-off values were defined at a significance level of * *p* ≤ 0.05, thus providing statistical evidence for an association between dose and clinical status (Figure 1). Significance was also assessed at ** *p* ≤ 0.01 to increase analytical rigor by limiting false-positive findings.

A comparison of the $\overset{-}{x}$ and SD of ZEN concentrations in the experimental groups with those noted in the control group (Figure 1) revealed two patterns. The first was observed in the RTCV, where ZEN levels increased over time during continued exposure. Although the proportion of statistically significant comparisons decreased over time, the data support the presence of a relationship between the ZEN dose and the clinical status of prepubertal gilts. The second pattern was observed in skeletal muscles, where ZEN levels varied. A dose-related association with the clinical status of gilts was observed only in group ZEN5 and on exposure dates D1 and D2, with a decreasing trend on D3 in all experimental groups.

### 3.4. Summary

The results demonstrate that ZEN accumulates in skeletal muscles and in the RTCV, indicating its ability to cross the blood–brain barrier. Consequently, ZEN disrupts neuroendocrine function, leading to alterations in endocrine homeostasis in the brain, which is regulated mainly by tanycytes as well as by interoceptive signals.

High COF values in skeletal muscles, which act as both hormone-sensitive and secretory organs, indicate that ZEN probably stimulates increased physical activity in young animals. This leads to enhanced muscle activity, which directly influences the function of the brain and other tissues.

High concentrations of ZEN were detected in the RTCV, which may have affected the clinical status of prepubertal gilts. In skeletal muscles, ZEN levels decreased over the course of the experiment, thus likely decreasing energy demand in prepubertal gilts.

## 4. Materials and Methods

### 4.1. Study Design

An in vivo experiment was performed at the Department of Veterinary Prevention and Feed Hygiene of the Faculty of Veterinary Medicine at the University of Warmia and Mazury in Olsztyn, Poland. The study was conducted on 60 clinically healthy prepubertal gilts obtained by crossing two domestic pig breeds (Polish Large White × Polish Landrace), with an initial body weight (BW) of 14.5 ± 2 kg [4]. The experiment lasted 6 weeks (42 days), during which the animals were housed in pens. They received diets that were identical in composition, and had unrestricted access to water. Gilts were randomly assigned to one of four groups: a control group (group C; n = 15) and three experimental groups (ZEN5, ZEN10, and ZEN15; n = 15 each) [67,68,69]. Groups ZEN5, ZEN10, and ZEN15 were orally administered ZEN (Sigma-Aldrich Z2125-26MG, St. Louis, MO, USA) at doses of 5 µg/kg BW, 10 µg/kg BW, and 15 µg/kg BW, respectively. Group C animals received a placebo. Analytical samples of ZEN were dissolved in 96 µL of 96% ethanol (SWW 2442-90, Polskie Odczynniki SA, Gliwice, Poland) in appropriate weight doses. Feed containing varying amounts of ZEN in an alcohol solution was placed in gel capsules, which were stored at ambient temperature before administration to allow the alcohol to fully evaporate. Zearalenone was administered in gel capsules on a daily basis prior to the morning feeding. Feed was used as the carrier medium, and group C pigs received the same gel capsules, but without the mycotoxin. Samples from the third ventricle of the brain, the longissimus dorsi muscle, and the quadriceps femoris muscle were collected on three dates (7, 21 and 42 days of exposure), immediately after slaughter. Each experimental group was housed in a separate pen within the same building. The pens had an area of 25 m^2^, thus complying with the relevant cross-compliance regulations (Regulation (EU) No 1306/2013 of the European Parliament and of the Council of 17 December 2013).

### 4.2. Experimental Feed

Gilts were weighed every week, and mycotoxin doses were calculated on an individual basis, depending on their BW [34,35,70]. All gilts received the same feed during the trial. The feed provided to all animals was supplied by a single producer. Pelleted feed was provided *ad libitum* twice daily, at 8:00 a.m. and 5:00 p.m., throughout the experiment. The composition of the complete diet, as declared by the manufacturer, is shown in Table 3 [34,35,70,71].

#### Preliminary Processing of Feed Samples

Feed samples were prepared in accordance with the applicable legislation (Commission Regulation (EU) No. 152/2009 of 27 January 2009, laying down the methods of sampling and analysis for the official control of feed, as amended). The quality of pelleted feed was verified during production using a quantitative method, including precise formulation of ingredients, thorough mixing, and conditioning. Standard methods (e.g., CIPAC MT 171, Collaborative International Pesticides Analytical Council Limited, Greece, https://share.google/4Qc7cBuKQghGGThLr, accessed on 6 May 2026) were applied to determine the dust content and particle size distribution of the final product (OECD 110, Organisation for Economic Cooperation and Development, Test No. 110: Particle Size Distribution, https://doi.org/10.1787/9789264069688-en).

The proximate chemical composition of the diets administered to pigs in groups C, ZEN5, ZEN10, and ZEN15 was determined using the NIRS™ DS2500 F feed analyzer (FOSS, Hillerød, Denmark), a monochromator-based NIR reflectance and transflectance analyzer with a scanning range of 850–2500 nm [3].

### 4.3. Determination of ZEN in Feed

Feed was analyzed for the presence of ZEN. The mycotoxin’s concentrations were determined by separation in immunoaffinity columns (Zearala-TestTM Zearalenone Testing System, G1012, VICAM, Watertown, MA, USA) and high-performance liquid chromatography (HPLC system, Agilent 1260, San Francisco, CA, USA)–mass spectrometry (MS, Agilent 6470, San Francisco, CA, USA) and chromatographic columns (Atlantis T3 3 μm 3.0 150 mm Column No. 186003723, Waters, AN Etten-Leur, Ireland). Zearalenone was separated using a mobile phase of acetonitrile:water:methanol (46:46:8, *v*/*v*/*v*) at a flow rate of 0.4 mL/min. The limit of quantitation (LOQ) was 2 ng/g for ZEN. Zearalenone and its metabolites were quantified at the Department of Veterinary Prevention and Feed Hygiene [3].

### 4.4. Toxicological Analysis of RTCV, Longissimus and Quadriceps Muscle Tissues

Five prepubertal gilts from each group were euthanized via intravenous injection of pentobarbital sodium (Fatro, Ozzano Emilia BO, Italy), followed by bleeding, on the following analytical dates: 1 (D1—exposure day 7), 2 (D2—exposure day 21), and 3 (D3—exposure day 42). Immediately after cardiac arrest, tissue samples were collected from the following anatomical regions: RTCV—samples (1.5 g) of dissected brain tissue obtained from the dorsal region of the third ventricle, including the choroid plexus and adjacent periventricular structures; the *longissimus* muscle—1 cm^3^ of tissue at the level of the last thoracic vertebra; and the *quadriceps* muscle—1 cm^3^ of tissue between the knee and hip joints. The collected samples were rinsed with phosphate buffer, prepared for analysis, and stored at a temperature of −20 °C.

#### 4.4.1. Extraction Procedure

Analyses of ZEN, α-ZEL, and β-ZEL in tissue samples were performed in accordance with the previously described protocol [3].

Zearalenone, α-ZEL, and β-ZEL were extracted from tissue samples using immunoaffinity columns (Zearala-TestTM Zearalenone Testing System, G1012, VICAM, Watertown, MA, USA), in accordance with the manufacturer’s instructions. The eluents were placed in a water bath at 50 °C, and were evaporated under a stream of nitrogen. The dry residue was stored at −20 °C until chromatographic analysis. The procedure was monitored using internal standards, and the results were validated by mass spectrometry.

#### 4.4.2. Chromatographic Quantification of ZEN and Its Metabolites

Zearalenone and its metabolites were quantified at the Institute of Dairy Industry Innovation in Mrągowo, Poland. The biological activity of ZEN, α-ZEL and β-ZEL in the examined tissues was determined by combined separation methods, using immunoaffinity columns (Zearala-TestTM Zearalenone Testing System, G1012, VICAM, Watertown, MA, USA), the Agilent 1260 liquid chromatography (LC) system, and a mass spectrometry (MS, Agilent 6470, Santa Clara, CA, USA) system. Samples were analyzed on a chromatographic column (Atlantis T3, 3 µm 3.0 × 150 mm, column No. 186003723, Waters, AN Etten-Leur, Ireland). The mobile phase consisted of 70% acetonitrile (LiChrosolvTM, No. 984 730 109, Merck-Hitachi, Mannheim, Germany), 20% methanol (LiChrosolvTM, No. 1.06 007, Merck-Hitachi, Mannheim, Germany), and 10% deionized water (MiliporeWater Purification System, Millipore S.A. Molsheim, France), to which 2 mL of acetic acid was added per 1 L of the mixture. The flow rate was 0.4 mL/min, and the oven column temperature was maintained at 40 °C. The column was flushed with 99.8% methanol (LIChrosolvTM, No. 1.06 007, Merck-Hitachi, Mannheim, Germany) to remove the bound mycotoxin. The chromatographic analysis was completed within 4 min.

Mycotoxin concentrations were determined using an external standard and subsequently expressed in ng/g. In order to guarantee the precision of the quantification process, matrix-matched calibration standards were used to reduce the impact of potential matrix effects, which could affect sensitivity. The calibration standards were dissolved in matrix samples according to the procedure that was used to prepare the remaining samples. The material used for the calibration standards was free of mycotoxins. The LOD for ZEN, α-ZEL and β-ZEL was defined as the concentration at which the signal-to-noise ratio (SNR) decreased to 3. The concentrations of ZEN, α-ZEL and β-ZEL were determined in each group and on three analytical dates (Table 3).

#### 4.4.3. Mass Spectrometric Conditions

The electrospray ionization (ESI) mass spectrometer was operated in negative ion mode. The parameters for MS/MS analysis were optimized for each compound. Linearity was assessed by constructing a calibration curve with six levels. The optimized conditions for analyzing the examined mycotoxins are presented in Table 4.

#### 4.4.4. Carryover Factor (COF)

Carryover toxicity is defined as a phenomenon in which an organism is capable of surviving exposure to low doses of mycotoxins. Mycotoxins can compromise tissue and organ function [71,72] and modify biological activity [73]. The COF was determined in the examined tissues when the daily dose of ZEN (5, 10 or 15 µg ZEN/kg BW) administered to each animal was equivalent to 560–32,251.5 µg ZEN/kg of the complete diet, depending on the animal’s daily feed intake. Mycotoxin concentrations in the tissues were expressed relative to the dry matter content of the samples.

The COF was calculated as follows:

COF = toxin concentration in tissue [ng/g] / toxin concentration in diet [ng/g]

#### 4.4.5. Statistical Analysis

The results were processed at the Department of Discrete Mathematics and Theoretical Computer Science at the Faculty of Mathematics and Computer Science of the University of Warmia and Mazury in Olsztyn, Poland. The bioavailability of ZEN and its metabolites in the RTCV and selected muscles of prepubertal gilts was analyzed in three experimental groups and in the control group, on different sampling dates. The results were expressed as $\overset{-}{x}$ and SD. The following parameters were included in the analysis: (i) differences between the mean values in three experimental groups (which received different doses of ZEN) and the control group were analyzed at three exposure dates; (ii) differences between the mean values within the groups (which received an identical dose of ZEN) were analyzed at each exposure date. In both tests, the differences between the means were estimated by one-way analysis of variance (ANOVA). If the differences between the groups were statistically significant, the differences between pairs of means were assessed by Tukey’s multiple comparison test. If all values in any of the groups were below the LOD (i.e., if the mean and variance were equal to zero), one-way ANOVA was performed on the values in the other groups, and the differences between the means in these groups were then compared with the difference in the population means (a difference of zero) using Student’s *t*-test. Student’s *t*-test was also used to assess the differences between the groups. The results of each analysis were considered to be highly significant at *p* < 0.01 (**) and significant at 0.01 < *p* < 0.05 (*). Data were analyzed using Statistica v.13 software (TIBCO Software Inc., Silicon Valley, CA, USA, 2017).

## 5. Conclusions

The study investigated whether low doses of ZEN administered *per os* to prepubertal gilts for six weeks affected ZEN, α-ZEL, and β-ZEL levels, as well as COF values in the RTCV and selected skeletal muscles. The results indicate the following:

1. Zearalenone was present in the analyzed tissues, whereas its metabolites were not detected.
2. The presence of ZEN in the RTCV confirms that it crosses the blood–brain barrier and may therefore participate in the hormonal homeostasis of the brain.

## Figures and Tables

**Table 1:** Mean values and standard deviations of ZEN, α-ZEL, and β-ZEL concentrations (ng/g) in the RTCV, *longissimus* and *quadriceps* muscles of prepubertal gilts.

Exposure Dates	Feed Intake [kg/day]	Total ZEN Doses in Groups [µg/kg BW]	Tissue	Group ZEN5 [ng/g]	Group ZEN10 [ng/g]	Group ZEN15 [ng/g]	Group C [ng/g]
**ZEN**							
**D1**	0.8	80.5/161.9/242.7	RTCV	0.26 ± 0.15	0.32 ± 0.23	0.34 ± 0.20	0
			*Longissimus*	0.66 ± 0.62	3.72 ± 2.04	5.11 ± 2.93 *	0
			*Quadriceps*	1.30 ± 0.29	0.52 ± 0.56	1.22 ± 1.12	0
**D2**	1.1	101.01/196.9/298.2	RTCV	0.35 ± 0.21	0.31 ± 0.17	0.43 ± 0.13	0
			*Longissimus*	0.74 ± 0.25	2.21 ± 2.08	4.63 ± 2.84 *	0
			*Quadriceps*	0.59 ± 0.33	1.04 ± 0.98	1.01 ± 0.76	0
**D3**	1.6	128.3/481.4/716.7	RTCV	0.45 ± 0.10	0.38 ± 0.04	0.31 ± 0.18	0
			*Longissimus*	0.42 ± 0.42	2.84 ± 2.92	3.26 ± 3.04	0
			*Quadriceps*	2.22 ± 2.79	0.22 ± 0.26	0.47 ± 0.54	0
**α-ZEL and β-ZEL**							
**D1** **–** **D3**	**Not applicable**			0			

**Abbreviations**: RTCV—roof of the third cerebral ventricle; D1—exposure day 7; D2—exposure day 21; D3—exposure day 42. Experimental groups: Group ZEN5—5 µg ZEN/kg BW; Group ZEN10—10 µg ZEN/kg BW; Group ZEN15—15 µg ZEN/kg BW; Group C (control, no ZEN exposure)—0 µg ZEN/kg BW. Differences were considered statistically significant at * *p* ≤ 0.05; notable difference between group ZEN5 and group ZEN15 on exposure dates D1 and D2.

**Table 2:** Carry-over factor values of ZEN from feed into selected tissues at different exposure doses.

COF				
Exposure Date	Tissue	Group ZEN5	Group ZEN10	Group ZEN15
**D1**	**RTCV**	3 × 10^−6^	1 × 10^−6^	1 × 10^−6^
	***Longissimus***	8 × 10^−6^	2 × 10^−5^	2 × 10^−5^
	***Quadriceps***	1 × 10^−5^	3 × 10^−6^	5 × 10^−6^
**D2**	**RTCV**	3 × 10^−6^	1 × 10^−6^	1 × 10^−6^
	***Longissimus***	7 × 10^−6^	1 × 10^−5^	1 × 10^−5^
	***Quadriceps***	5 × 10^−6^	5 × 10^−6^	3 × 10^−6^
**D3**	**RTCV**	3 × 10^−6^	7 × 10^−7^	4 × 10^−7^
	**Longissimus**	3 × 10^−6^	5 × 10^−6^	4 × 10^−6^
	**Quadriceps**	1 × 10^−5^	4 × 10^−7^	6 × 10^−7^

**Abbreviations:** COF—carry-over factor; RTCV—roof of the third cerebral ventricle; D1—exposure day 7; D2—exposure day 21; D3—exposure day 42. Experimental groups: Group ZEN5—5 µg ZEN/kg BW; Group ZEN10—10 µg ZEN/kg BW; Group ZEN15—15 µg ZEN/kg BW.

**Table 3:** Ingredient composition of the complete diet.

Ingredient	Manufacturer-Declared Composition (%)
Soybean meal	16
Wheat	55
Barley	22
Wheat bran	4.0
Limestone	0.3
Zitrosan	0.2
Vitamin–mineral premix ^1^	2.5

^1^ Composition of the vitamin–mineral premix per kg: vitamin A—500,000 IU; Fe—5000 mg; vitamin D_3_—100,000 IU; Zn—5000 mg; vitamin E (alpha-tocopherol)—2000 mg; Mn—3000 mg; vitamin K—150 mg; Cu (CuSO_4_·5H_2_O)—500 mg; vitamin B_1_—100 mg; Co—20 mg; vitamin B_2_—300 mg; iodine—40 mg; vitamin B_6_—150 mg; Se—15 mg; vitamin B_12_—1500 μg; L-lysine—9.4 g; niacin—1200 mg; DL-methionine + cystine—3.7 g; pantothenic acid—600 mg; L-threonine—2.3 g; folic acid—50 mg; tryptophan—1.1 g; biotin—7500 μg; phytase + choline—10 g; ToyoCerin probiotic + calcium—250 g; antioxidant + mineral phosphorus and released phosphorus—60 g; Mg—5 g; Na and Ca—51 g.

**Table 4:** Optimized analytical parameters for achieving the highest detection efficiency, accuracy and sensitivity of ZEN and its metabolites [3,28].

Analyte	Precursor	Quantification Ion	Confirmation Ion	LOD (ng mL^−1^)	LOQ (ng mL^−1^)	Linearity (%R^2^)
**ZEN**	317.1	273.3	187.1	0.03	0.1	0.999
**α-ZEL**	319.2	275.2	160.1	0.3	0.9	0.997
**β-ZEL**	319.2	275.2	160.1	0.3	1	0.993

## Data Availability

The original contributions presented in this study are included in the article. Further inquiries can be directed to the corresponding author.
